# Impact of depressive tendency on intertemporal decision-making in college students: the moderated mediation effects of perceived stress and self-control

**DOI:** 10.3389/fpsyt.2025.1625795

**Published:** 2026-01-12

**Authors:** Yutong Xie, Zhen Wang, Danning Su, Yuhang Li, Xinyu Gao, Jing Han, Bin Xie

**Affiliations:** 1 Graduate Department, Xi’an Physical Education University, Xi’an, China; 2 Department of Psychology, Xi'an Physical Education University, Xi’an, China; 3 Student Mental Health Center, Student Affairs Office, Xi'an Physical Education University, Xi’an, China

**Keywords:** college students, depressive tendency, intertemporal decision-making, perceived stress, self-control

## Abstract

**Background:**

This study explored how depressive tendencies influence college students’ intertemporal decision-making, focusing on perceived stress and self-control as potential mechanisms to inform intervention strategies.

**Methods:**

An online survey using CES-D, Perceived Stress Questionnaire, College Student Self-Control Scale, and Intertemporal Decision-making Scale collected 1,469 responses, with 436 valid ones from students scoring >10 on depressive tendency. Data were analyzed via descriptive, correlation, regression, and moderated mediation tests.

**Results:**

Results showed depressive tendency positively correlated with intertemporal decision-making (r=0.55, p<0.01), positively predicted perceived stress (r=0.64, p<0.01), and negatively predicted self-control (r=-0.64, p<0.01). It directly predicted intertemporal decision-making (direct effect=0.23, 95% CI [0.07, 0.16]) and indirectly through perceived stress and self-control (indirect effect=0.29), forming a “depressive tendency - perceived stress - self-control - intertemporal decision-making” model.

**Conclusions:**

Depressive tendencies drive preference for immediate small rewards via heightened perceived stress and impaired self-control, suggesting interventions should focus on stress management and self-control training.

## 1 Introduction

With the intensification of social competition and the accelerated pace of life, college students experience gradually heavy psychological pressure nowadays. There has been a massive number of college students with depressive tendencies, and this trend is on the rise year by year, highlighting the urgency and importance of paying attention to the mental health issues of college students. Depressive tendency, described for the first time by Judd et al. in 1994, is a sub-threshold depressive state where an individual, not yet up to the diagnostic criteria for depression, exhibits two or more depressive symptoms for at least two weeks (1). However and critically, individuals may fall into negative emotions for a long time in case of long-term presence of this experience, failing to obtain happiness and a sense of control, gradually losing cognitive and adaptive functions. Eventually, this group of students may develop depression, causing serious adverse impact on their physical and mental health consequently.

It has been documented that depressive tendency plays a role in the intertemporal decision-making behavior of an individual. Intertemporal decision-making refers to a process that an individual determine his/her preferences after weighing their gains or losses at different time points (2). This decision-making behaviors, which are extremely common, can be found everywhere and every time in our daily life, such as when choosing and determining between immediate consumption or savings for greater future returns. Critically, time discounting is a key feature in this process, which may indicate the underestimation of future gains-losses and prioritization of immediate needs (3). This tendency may result in an individual’s pursuit for short-term satisfaction by sacrificing long-term well-being (4). Based on the calculation through Mazur’s hyperbolic model, this rate of time discounting can be measured by using the mathematical expression of “V=1+kDA,” where “V” is a subjective value, “A” refers to the delay amount, “D” is the delay days, and “k”represents the time discounting rate. Individuals with higher “k” value may tend to instant gratification (5, 6). Intertemporal decision-making occupies a key position for college students’ psychological development. Enhancement in this ability can contribute to their learning of weighing the pros and cons, restraining impulses, and cultivating delayed gratification when dealing with short-term temptations and long-term goals. In addition, it can benefit the cultivation of risk awareness and coping ability, allowing students to strength their psychological resilience.

But how does depression affect the decision-making behavior of college students? According to the existing research, college students with depressive tendencies tend to overestimate risks and underestimate benefits, especially in situations involving social and intimate relationships (7). For example, they are more hesitant in making decisions such as whether to actively make new friends or participate in group activities, which exacerbates their sense of social isolation (7). At the same time, some researchers have found that individuals with depressive tendencies exhibit a higher time discounting rate in decision-making, in other words, more apparent preference of immediate rewards (8). Moreover, individuals with negative emotions tended to underestimate future rewards in intertemporal decision-making (8), possibly due to pessimistic expectations of the future and excessive emphasis on immediate rewards (6). Individuals with depressive tendencies may feel anxious about the uncertainty of the future, and therefore tend to choose rewards that can be obtained immediately to reduce the psychological pressure caused by uncertainty (9). With respect to the above, when making decisions, the presence of depressive tendencies tends to encourage individuals to choose immediate gratification over future high returns. Such a pattern of decision-making not only weakens an individual’s ability to withstand pressure, but also hinders the development of psychological resilience. Therefore, in-depth exploration of the underlying mechanisms by which depressive tendencies affect intertemporal decision-making is of great significance for blocking this negative impact.

Recent research evidence supports that intertemporal decision-making can be influenced by multiple factors, including not only depressive emotions, but also perceived stress and self-control. Perceived stress, as proposed by Lazarus, is a subjective experience of an individual in response to stressful events, with its intensity and impact varying depending on the cognition and feelings of a specific individual (10). Under high-stress conditions, individuals may prefer immediate rewards to alleviate psychological stress, resulting in higher time discounting rates. Moreover, as reported, the perceived stress of adolescents exhibits a significant negative correlation with intertemporal decision-making, and adolescents may be more inclined to choose timely gratification under stress when making decisions (11). In addition, depressive tendencies and perceived stress may interact with each other, exhibiting a joint influence in the process of intertemporal decision-making. Especially, given the gradually increased number of individuals with high levels of depression tendencies and perceived stress among college students (12), it is pivotal to emphasize on the dual effects of the two factors on intertemporal decision-making.

Self-control ability can also affect intertemporal decision-making. It enables individuals to pursue long-term goals by consciously suppressing their instinctual responses (13). Thaler and Shefrin proposed that in individuals with self-control ability, they could suppress short-term preferences to choose long-term goals when there was a conflict between short-term outcomes and long-term goals in intertemporal decision-making (Thaler & Shefrin, (14)).

Individuals with high self-control were more likely to make delayed choices, while those with low self-control preferred immediate decisions (15). Besides, the level of self-control of resources can effectively measure self-control ability, and subjects with decreased self-control of resources were reported to prefer immediate choice in intertemporal decision-making potentially (16).

Systematic review of prior studies suggests a significant mutual influence relationship between perceived stress and self-control. However, the dominated impact of perceived stress on self-control ability has been confirmed by existing research. Theoretically, the strength model of self-control, proposed by Baumeister, indicates that the exercise of self-control depends on limited resources, which, similar to muscles, can become fatigued after exertion. Self-control behavior can cause short-term damage in subsequent self-control tasks, i.e., the resource depletion hypothesis of self-control (13, 17). On the basis of this hypothesis, Vohs et al. found that there would be significant consumption of internal resources required for executive function when participants were in high stress situations, thereby significantly weakening their subsequent self-contro (18). Simultaneously, Hou Caili proposed the effect of perceived stress on the intertemporal decision-making of individuals, and self-control might play a partial mediation role in this process (19). In another research carried out by Yueli Zheng, perceived stress was found to affect life satisfaction, and self-control could mediate this process partially (20). Overall, a research consensus has been reached regarding the unidirectional impact mechanism of perceived stress on self-control in this field. It provides important theoretical basis and practical direction for subsequent intervention research, such as training the self-control ability of an individual through stress management.

In view of the above, intertemporal decision-making, as an important cognitive process for individuals to weigh gains or losses at different time points, has a profound impact on the psychological development of college students. Moreover, depressive tendency, perceived stress, and self-control are three key psychological factors influencing intertemporal decision-making. As a result, this study explored the underlying psychological mechanisms by which depressive tendency affect intertemporal decision-making. Significantly, in addition to providing theoretical basis for promoting the mental health development of college students with depressive tendencies, findings in our study may also offer practical guidance for improving college students’ decision-making abilities.

## 2 Materials and methods

### 2.1 Participants

Participants from three universities in Zhejiang, Shaanxi, and other regions of China, were surveyed using questionnaires supported by an online survey platform Wenjuanxing (www.wjx.cn). Through convenient sampling, data were collected through campus visits and based on online sharing of the questionnaire link. Students participated voluntarily in the survey by clicking on the link or scanning the QR code to fill out the questionnaire. All stored data from this anonymous survey were kept confidential. With the collection of 1,469 questionnaires, 436 valid questionnaires remained after removing questionnaires with issues such as missing answers, unchanged answers, and answers within a short period of time, with depressive tendency scores defined as > 10 points. Participants aged 20.4 years old (± 1.31) on average. In view of the final samples, there were 208 male participants (47.7%) and 228 female participants (52.2%). Besides, the specific numbers (proportions) of freshmen, sophomores, juniors and seniors were 87 (19.9%), 135 (30.9%), 119 (27.2%), and 95 (21.7%), respectively (**Table 1**).

**Table 1:** Distribution of basic information of the survey subjects.

Demographic variables	Category	N	Percentage (%)
Gender	Male	208	47.70
	Female	228	53.27
Grade	Freshmen	87	20.33
	Sophomore	135	31.54
	Junior	119	27.80
	Senior	95	22.20

### 2.2 Hypothesis of this study

Hypothesis I: Depressive tendency could significantly affect the intertemporal decision-making of college students. College students with higher levels of depression tended to choose immediate small rewards (i.e. higher time discounting rates) during intertemporal decision-making, exhibiting stronger immediate preferences.

Hypothesis II: Perceived stress could mediate the relationship between depressive tendency and intertemporal decision-making. Depressive tendency could increase college students ‘ levels of perceived stress, thereby promoting their preference for immediate rewards in intertemporal decision-making.

Hypothesis III: Self-control could also mediate the relationship between perceived stress and intertemporal decision-making, forming a moderated mediation effect. Beyond a direct impact on intertemporal decision-making through perceived stress, depressive tendency could also indirectly reinforce immediate decision-making preferences by weakening self-control abilities.

### 2.3 Sample size adequacy

To define the required adequate sample size, G ∗ Power (3.1.9.7) was used for *post-hoc* efficacy analysis. Under a given condition of D = 0.3 and α =0.05 (medium effect size), the calculated statistical power (1-β) was 0.95 for the 436 sample size in this study, supporting the adequacy of sample size in this study.

### 2.4 Testing tools

#### 2.4.1 Center for Epidemiological Studies Depression Scale Short Form

The CES-D consists of 9 items, each rated on a 4-point scale. Based on samples from various age groups across the country, the calculated reliability and test-retest reliability were 0.85-0.88 and 0.49, respectively, with the item-total score correlation ranged between 0.51-0.69. The criterion validity associated with the CES-D was 0.61 (P<0.001), and the score of depression diagnosed at the first visit was significantly higher than that of depression diagnosed with medication (t=4.76, P<0.001). According to previous research on the Chinese college students, the total score of the CES-D correlated highly with that of the original 20-item version, ranging from 0.94 to 0.96. In CES-D, the depressive tendency and high-risk depression were determined at the threshold of 10 points and 17 points, respectively (20). The Cronbach’s alpha of CES-D was 0.85 in the present study.

#### 2.4.2 Perceived Stress Questionnaire

A Perceived Stress Questionnaire (Chinese version), originally developed by Sheldon et al. and revised by Yang Tingzhong and Huang Hanteng, was used in this study. For measuring the perceived stress in the past month, this questionnaire consists of 10 items, each rated on a 5-point Likert scale. A higher score indicates more perceived stress. According to previous measurements on Chinese college students, the Cronbach’s coefficient of this questionnaire was 0.78. The average inter-item correlation was 0.28, and item-total score correlation ranged from 0.37 to 0.53, indicating high homogeneity and internal consistency (21). Similarly, the Cronbach’s alpha was calculated to be 0.94 in our study.

#### 2.4.3 College Student Self-Control Scale

The Chinese Version of the College Students’ Self-Control Scale, revised by Tan Shuhua et al., was used in this study. The revised scale scored college students from five dimensions of impulse control, healthy habits, resistance to temptation, focus on work, and entertainment moderation, with 19 items totally. Each item was rated on a 5-point Likert scale, with greater self-control abilities determined when there were higher total scores. According to previous experimental results targeting Chinese university students, corresponding internal consistency reliability and test-retest reliability were 0.862 and 0.850, respectively (22). Good reliability and validity were found based on theCronbach’s alpha value of 0.83 in this study.

#### 2.4.4 Intertemporal Decision-making Scale

This study employed an intertemporal decision-making experimental paradigm with reference from the research of Wang and Dvorak in 2010. The research paradigm consists of 14 questions, and the first 7 and last 7 questions can be considered parallel tests. In this study, the first 7 questions were selected as the formal experimental measurement tool for the matching task. In the scale, participants can choose to receive a smaller monetary reward (non-fixed) tomorrow or a larger reward (non-fixed) after a certain delay. The “k” value corresponding to each question was determined according to the equation of the hyperbolic model, where”Afuture”and”Acurrent”represent the future and current reward amount, respectively. “D” represents the delay time (in days, months, or years). “K” represents the time discounting rate, which is a reflection of the preference of an individual for delayed rewards. In other words, individuals with higher or lower “ k “ may reveal a stronger preference for smaller rewards in the present, or a greater willingness of waiting for larger rewards in the future. After answering each question by the subjects in sequence, this study would sort the “k” values from small to large to determine the inflection point where the subjects’ decisions changed (23). Noticeably, data with only one inflection point were eligible for each, and those exceeding one inflection point were invalid that should be deleted from this study.

For example, in the question “Receive ¥100 tomorrow or ¥320 after 365 days”, D (delay time)=365, A (future amount)=320, and A (current amount)=100, then k=≈0.006. If the participant chose “larger future reward” in the first three questions, but “smaller immediate reward” starting from question 4, then question 4 would be the inflection point.

### 2.5 Data analysis

Data analyzed in this study were sourced from anonymous self-report questionnaires, with potential common methodological biases. Data analysis was conducted in SPSS 26.0. The approximate normal distribution of data was confirmed by data normality test, with both skewness and kurtosis <1. The analysis was finished in three steps: (1) summary of demographic information through descriptive statistics; (2) correlation analysis to clarify the relationship of depressive tendency, perceived stress, self-control, and intertemporal decision-making; and (3) validation of inter-variable relationship through regression analysis and SPSS Macro PROCESS.

## 3 Results

### 3.1 Testing of common method bias (CMB)

All data were obtained from self-assessment questionnaires. To confirm the existence of CMB, an unrotated exploratory factor analysis was conducted on all items within the questionnaire by Harman’s single-factor test. Finally, there were 12 common factors with eigenvalues >1, of which the top-1 accounted for 25.30% of the total, smaller than the criterion of 40% proposed by Podsakoff et al. Therefore, our study revealed no serious CMB.

### 3.2 Demographic characteristics

At a threshold value of 10 points for depressive tendency regarding the total score of the CES-D, 17 points was defined as the high-risk score for depression. In our study, 436 college students had high risk of depression. **Table 2** presents the descriptive statistics of all variables based on the perceived stress, self-control, and intertemporal decision-making scores of all participants according to their different demographic characteristics.

**Table 2:** Descriptive statistics and demographic differences of variables.

Variables		N	M	SD	t	F	P
Depressive Tendency	Male	208	26.63	2.38 (±0.17)	0.74	0.42	0.52
	Female	228	26.47	2.41 (±0.16)			
	Freshmen	87	26.93	2.16 (±0.23)		0.97	0.41
	Sophomores	135	26.04	2.49 (±0.22)			
	Juniors	119	26.71	2.29 (±0.21)			
	Seniors	95	26.73	2.48 (±0.26)			
Perceived Stress	Male	208	39.25	3.20 (±0.22)	0.87	0.06	0.8
	Female	228	29.53	3.49 (±0.23)			
	Freshmen	87	26.93	2.16 (±0.23)		0.42	0.74
	Sophomores	135	26.04	2.49 (±0.22)			
	Juniors	119	26.71	2.29 (±0.21)			
	Seniors	95	26.73	2.48 (±0.26)			
Self-control	Male	208	45.49	4.82 (±0.33)	-0.54	1.25	0.27
	Female	228	45.73	4.52 (±0.30)			
	Freshmen	87	44.89	3.49 (±0.37)		0.52	0.67
	Sophomores	135	45.81	4.52 (±0.30)			
	Juniors	119	45.75	4.94 (±0.45)			
	Seniors	95	45.85	3.91 (±0.40)			
Intertemporal Decision-making	Male	208	1.47	0.20 (±0.01)	1.25	0.01	0.98
	Female	228	1.44	0.18 (±0.01)			
	Freshmen	87	1.48	0.18 (±0.02)		1.27	0.28
	Sophomores	135	1.47	0.17 (±0.01)			
	Juniors	119	1.45	0.19 (±0.02)			
	Seniors	95	1.45	0.22 (±0.02)			

No significant differences were noticed in the indices of depressive tendency, perceived stress, self-control, and intertemporal decision-making among the four academic years and between genders. Therefore, the surveyed college students exhibited high degree of homogeneity in psychological characteristics and decision-making behaviors. During this relatively special period of studying in university stage, students may experience similar academic pressure, social environments, and life rhythms. These common external factors may results in a tendency of consistent psychological state and behavioral performance, thereby masking differences between genders and among grades.

### 3.3 Analyses of correlations among depressive tendency, perceived stress, self-control, and intertemporal decision-making

By employing a Pearson correlation analysis, this study continued to clarify correlations among perceived stress, self-control, and intertemporal decision-making for college students with depressive tendencies. The results are shown in **Table 3**.

**Table 3:** Correlational relationships among variables.

Variable	M	SD	Depressive tendency	Perceived stress	Self-control	Intertemporal decision-making
Depressive tendency	26.18	3.16	1	6.40E+00	-.65^**^	.55^**^
Perceived Stress	37.55	7.37		1	-.71^**^	.61^**^
Self-control	47.69	11.43			1	-.63^**^
Intertemporal Decision-making	1.46	0.19				1

*p<0.05; **p<0.01.

As shown in **Table 3**, depression tendency, perceived stress, and intertemporal decision-making were positively correlated with each other, suggesting that students with higher depression tendencies would have stronger immediate preference for perceived stress and intertemporal decision-making. In case of high stress of an individual, there would also be possible increase in the short-term choice tendency of depression tendency and intertemporal decision-making. Simultaneously, self-control was negatively correlated with depressive tendency, perceived stress, and intertemporal decision-making. In other words, students with stronger self-control ability would have lower levels of depressive tendency and perceived stress, and more inclined intertemporal decision-making towards long-term choices. On the contrary, weak self-control could easily lead to high depression, high stress, and a preference for immediate gratification.

### 3.4 Analysis on the moderated mediation effect

For subsequent investigation, this study used depression tendency as an independent variable, intertemporal decision-making as a dependent variable, as well as perceived stress and self-control as mediator variables. Model 6 in the plugin SPSS Macro PROCESS program provided by Hayes (2013) was utilized to conduct 5,000 self-sampling tests to examine the mediation effect. The results are shown in **Tables 4** and **5**.

**Table 4:** Regression analysis of the mediating model between perceived stress and self-control in depressive tendency and intertime decision-making.

Regression equation		Goodness of fit indexes			Coefficient significance	
Outcome variable	Predictive variables	R	R2	F	Beta	t
Intertemporal decision-making	Depressive tendency	0.56	0.31	191.28	0.55	13.83^**^
Intertemporal decision-making	Depressive tendency	0.65	0.41	154.56	0.28	5.66^**^
	Perceived stress				0.44	9.06^**^
Intertemporal decision-making	Depressive tendency	0.66	0.43	164.12	0.25	5.16^**^
	Self-control				-0.47	-9.77^**^
Intertemporal decision-making	Depressive tendency	0.47	0.68	125	0.16	3.20^**^
	Perceived stress				0.28	5.20^**^
	Self-control				-0.33	-6.23^**^

*p<0.05; ** p<0.01.

**Table 5:** Moderated mediation effect test.

Effect type	Mediation effect pathways	Mediation effect values	Boot SE	Boot LL	Boot UL
	Direct effect	0.23	0.1	0.07	0.16
0.Indirect effect	Path 1: Depressive Tendency—Perceived Stress— Intertemporal Decision-making	0.12	0.37	0.05	0.19
	Path 2: Depressive Tendency—Self-control—Intertemporal Decision-making	0.1	0.03	0.04	0.15
	Path 3: Depressive Tendency—Perceived Stress— Self-control—Intertemporal Decision-making	0.07	0.02	0.03	0.13

As presented in **Table 4**, depressive tendency could positively predict intertemporal decision-making (B = 0.55, t=3.20, p<0.0). In particular, depressive tendency still had a significant positive predictive effect on intertemporal decision-making even after incorporating the mediation variable of perceived stress (B = 0.28, t=5.66, p<0.01). After the incorporation of the mediator variable, i.e., self-control, depressive tendency still had a significant positive predictive effect on intertemporal decision-making (B = 0.25, t=5.16, p<0.01). Furthermore, when both perceived stress and self-control were considered as mediator variables, there remained significant positive predictive effect of depressive tendency on intertemporal decision-making (B = 0.16, t=3.20, p<0.01). Therefore, perceived stress and self-control might play partial mediation roles in the impact of depressive tendency on intertemporal decision-making.

**Figure 1:**
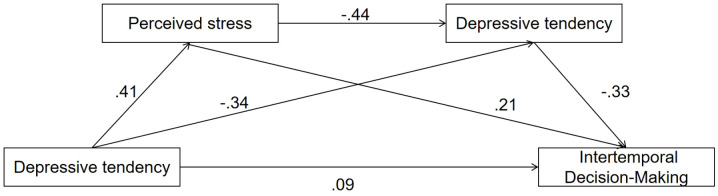
Moderated mediation model diagram.

From **Table 5**, the direct effect of depressive tendency on intertemporal decision-making was 0.23, with the confidence interval of (0.07, 0.16), excluding zero. Meanwhile, the overall effect of indirect effects was 0.29, and the 95% confidence intervals in paths 1–3 did not include 0, indicating that intertemporal decision-making could be directly predicted by depressive tendency, and that it could be predicted through the mediation effect of perceived stress and self-control. Collectively, perceived stress and self-control played partial mediation roles in the relationship between depressive tendency and intertemporal decision-making. **Figure 1** profiles the moderated mediation model diagram.

## 4 Discussion

In the present study, no significant differences were observed in indices for depressive tendency, perceived stress, self-control, and intertemporal decision-making in the surveyed college students across the four academic years and between genders, suggesting the involvement of multiple factors. It can be interpreted that the psychological characteristics of college students are subjected to the influence of diverse factors since they are in a critical stage of physical and mental development. For example, as suggested by prior research, the first year of college is an important turning point for students from middle school to university. These freshman may face many negative psychological experiences, such as confusion induced by unfamiliar environment, status gap and imbalance, contrast between university reality and ideals, maladjustment in interpersonal relationships, desire and variation in love, loss and hesitation in goals, etc. (24), leading to potential phenomenon of depression in these students. Meanwhile, sophomores and juniors may encounter with serious problems in academic pressure, disharmony in interpersonal relationships, confusion between ideals and reality, confusion about sex and emotions, and neurosis. In addition, seniors may deal with serious problems in employment, postgraduate entrance examination, etc. (25). Therefore, both male and female college students experience various pressures and conflicts in each grade, with one going against the other. There existed no significant differences in the four variables across the four grades and genders.

This study profoundly analyzed the intertemporal decision-making behavior of college students with depressive tendencies, revealing obvious negative correlation. In other words, individuals with depressive tendencies would be more prone to choosing immediate rewards with smaller returns, rather than delayed rewards with larger returns. In response to this phenomenon, some researchers have proposed the Self-Continuity Model, which explores individual decision-making behaviors through subjective time perception. In this theory, individuals with stronger sense of identification with their “future self” may be more willing to sacrifice current interests for future rewards (26). In contrast, individuals in negative emotional states may tend to detach from unhappy emotions, resulting in increased allocation of attentional resources to the current state (27), which leads to future rewards being perceived as “farther” psychologically, and thus these individuals will be more willing to choose immediate gratification (28). This theory has also been confirmed by brain imaging research. It has been revealed that there would be significantly enhanced functional connections between the anterior cingulate cortex, hippocampus, and amygdala in the brain when participants made predictable decisions for the future (29), while individuals with depression tendencies would have narrowed volume of the amygdala (30), leading possibly to a weakening of the aforementioned connectivity functions. In this regard, rather than affected by a single psychological variable, the “instant satisfaction” of college students with depressive tendencies in intertemporal decision-making may be influenced by multiple mechanisms, ultimately limiting their behavior in the present small benefits.

Furthermore, perceived stress and self-control was a pivotal mediated moderator between depressive tendencies and intertemporal decision-making. As reported previously, college students with depressive tendencies usually experienced higher perceived stress, which would further weaken their self-control in intertemporal decision-making (31). This perceived stress may be developed from academic pressure, interpersonal relationship pressure, future uncertainty and various other inducers. Due to the high perceived stress, students would prefer immediate gratification in decision-making to alleviate current psychological stress. Moreover, perceived stress would disturb students’ emotional state and cognitive assessment, resulting in more obvious tendency towards immediate rewards to alleviate their current psychological stress, resulting in a higher time discounting rate in intertemporal decision-making (25). Besides, college students with depressive tendencies would have suppressed self-control owing to their higher perceived stress. The decline in self-control may make individuals choose impulsively in case of temptations, manifesting as a preference for immediate rewards and a tendency toward immediate gratification (26). Moreover, in addition to compromising the performance of intertemporal decision-making, the deficiency of self-control may further exacerbate depressive tendencies, forming a vicious cycle.

## 5 Conclusions

(1) Depressive tendencies have a significant negative impact on college students ‘ intertemporal decision-making;

(2) Perceived stress and self-control exhibit moderated mediation effects in the intertemporal decision-making of college students with depressive tendencies.

## 6 Limitations and shortcomings

There are still limitations in this study, despite some promising results. Firstly, our study was carried out based on the inclusion of a group of college students; moreover, this study only conducted intra-group modeling for college students with depressive tendencies, with the lack of more geographical and group characteristics, and did not consider other student groups, leading to potentially compromised external validity of the results. More subjects from different regions and of different types can be enrolled in future research to strengthen the generalizability of the results. Secondly, with potential subjective biases, the psychological scales and questionnaire surveys used in this study might be impossible to fully capture the complexity of the actual decision-making. It necessitates the performance of in-depth study to more comprehensively evaluate relevant variables by integrating behavioral experiments, physiological indicators, etc. Additionally, this study adopted a cross-sectional design, where causal relationships between variables were affected, with the absence of validation of intervention measures. Subsequent study can design targeted psychological intervention programs to evaluate their effectiveness in improving the intertemporal decision-making ability of college students with depressive tendencies.

## Data Availability

The raw data supporting the conclusions of this article will be made available by the authors, without undue reservation.
